# Dual activities of lysis and capsule degradation enable the bacteriophage protein Lys33795 to effectively disrupt *Streptococcus suis* biofilms

**DOI:** 10.1371/journal.ppat.1014741

**Published:** 2026-10-23

**Authors:** Yalu Ji, Meimei Zhang, Liran Song, Hui Ning, Li Wang, Cao Qiu, Jingmin Gu

**Affiliations:** 1 State Key Laboratory for Diagnosis and Treatment of Severe Zoonotic Infectious Diseases, Key Laboratory for Zoonosis Research of the Ministry of Education, Institute of Zoonosis, and College of Veterinary Medicine, Jilin University, Changchun, China; 2 Jiangsu Co-Innovation Center for the Prevention and Control of Important Animal Infectious Diseases and Zoonoses, Yangzhou University, Yangzhou, China; 3 Yunnan Provincial Key Laboratory of Zoonosis Control and Prevention, Yunnan Institute of Endemic Diseases Control and Prevention, Kunming, China; Washington University School of Medicine in Saint Louis: Washington University in St Louis School of Medicine, UNITED STATES OF AMERICA

## Abstract

*Streptococcus suis* (*S. suis*) is an encapsulated zoonotic pathogen that can form recalcitrant biofilms. Bacteriophage-derived endolysins and depolymerases represent promising antibiofilm agents, but currently, no report has described protein which displays both of lytic and capsule degradation activities simultaneously. Here, we mined the porcine gut virome and identified Lys33795, a dual-activity antibacterial protein that degrades both capsule and peptidoglycan of *S. suis*. Lys33795 consists of an N-terminal GH25_muramidase domain and a C-terminal cell wall binding domain (CBD). However, translational reinitiation at an internal start codon (M_217_) in the Lys33795 sequence generates an additional CBD that may assembles with the full-length protein to form an oligomeric complex. Interestingly, the GH25 muramidase truncation fragment of Lys33795 not only retains its classical function as a peptidoglycan hydrolase for degrading bacterial cell walls but also exhibits degradation activity towards capsule. This discovery contrasts sharply with the traditional functional understanding of the GH25 family. In addition, the CBD alone has a stable dimer and, as a nonstandard depolymerase without any classical polysaccharide-degrading catalytic domains, retains its degradation activity against *S. suis* capsule. We elucidated the molecular mechanism of Lys33795 exhibiting dual activity by combining mutagenesis, lysis, and capsule degradation activity assays. In addition, the results of *in vitro* and *in vivo* experiments showed that Lys33795 can effectively prevent and clear *S. suis* biofilms, and is more effective than single active mutants, and has a enhance effect when used in combination with antibiotics. This study describes the first multimeric bacteriophage protein with dual activities of lysis and capsule degradation against *S. suis*, encoded by a single gene. These findings provide valuable insights for designing potent antibacterial proteins targeting encapsulated pathogens.

## Author summary

Some pathogenic bacteria are protected by their surface capsules and can form a sticky biofilm as a shelter, preventing being eliminated by antibiotics and other drugs. *S. suis* is such a zoonotic pathogen. In this study, we discovered a novel bacteriophage antimicrobial protein Lys33795, whose multimeric structure is encoded by a single gene and possesses the dual degradation function of capsule and cell wall peptidoglycan. Such a dual-functional antimicrobial protein has never been observed before. Interestingly, both of the separate GH25_muramidase domain and the separate CBD of Lys33795 exhibit degradation activity towards capsule, which is in stark contrast to the traditional functions understanding of the GH25 family and CBD domain. The experimental results showed that this protein could kill various serotypes of *S. suis*. Whether it is preventing biofilm formation or clearing existing biofilms, its performance is superior to that of single lytic or capsule degradation activity mutant protein. This discovery provides a new approach for developing novel antibacterial drugs and also provides new insights for the prevention and control of other capsular Gram-positive bacteria.

## Introduction

*Streptococcus suis* (*S. suis*) is a major gram-positive encapsulated zoonotic pathogen that causes severe invasive diseases such as meningitis, septicaemia, and streptococcal toxic shock-like syndrome in both swine and human [1,2]. *S. suis* can be classified into 29 serotypes based on the capsular polysaccharide (CPS) type, among which serotype 2 (SS2) is the most virulent and poses a significant threat to the global pig industry and public health [1]. A key virulence trait of *S. suis* is its ability to form dense biofilms, wherein bacterial cells are encased in a self-produced extracellular matrix rich in CPS and other polysaccharides [3,4]. This biofilm architecture protects the embedded bacteria from antibiotic penetration and host immune clearance, and it facilitates the horizontal transfer of antimicrobial resistance genes, thereby contributing to persistent, difficult-to-cure infections and therapeutic failure [5,6]. In addition, the increasing prevalence of multidrug-resistant *S. suis* strains, with most clinical isolates showing resistance to tetracyclines and macrolides, further highlights the urgent need for alternative therapeutic strategies that can effectively target biofilm-encapsulated bacteria [7,8].

Bacteriophage-encoded antimicrobial proteins, particularly endolysins (lysins) and depolymerases, have emerged as promising alternatives to conventional antibiotics [9,10]. Lysins are hydrolytic enzymes that specifically recognize the host cell wall and cleave the peptidoglycan to digest the bacterial cell wall [11]. Depolymerases specifically target and degrade CPS and extracellular polysaccharides, thereby disrupting the biofilm matrix and sensitizing embedded bacteria. Both classes of enzymes have shown efficacy in removing bacterial biofilms and *in vivo* therapy [9]. However, to date, no bacteriophage -encoded depolymerase specifically targeting the *S. suis* CPS has been identified, and the bacteriophage-derived protein that possesses both intrinsic lytic and CPS depolymerase or capsule degradation activities against *S. suis* remains unreported. Given that CPS is both a major virulence factor and a structural component of the biofilm matrix, a dual-activity protein that is capable of simultaneously degrading the capsule shield and killing the exposed bacteria would represent a superior strategy for biofilm eradication and infection control.

Generally, lysins possess a modular architecture comprising one or more catalytic domains (CDs) and a cell wall-binding domain (CBD) that confers target specificity, which are interconnected by a short flexible linker [12]. Catalytic domains are responsible for the cleavage of peptidoglycan bonds within the cell wall and can be classified into four groups: N-acetylmuramoyl-L-alanine amidases, N-acetylglucosaminidases, lysozymes, and endopeptidases [13–15]. CBDs display variety and distinguish discrete epitopes present within the host cell wall, such as carbohydrates or teichoic acids, thus giving rise to species-specific or strain-specific activity of a particular lysin [16,17]. Bacteriophage depolymerases are typically large trimeric proteins with multiple structural domains. A typical trimeric depolymerase has a molecular weight of approximately 200 kDa and consists of three main domains: a flexible N-terminal domain; a right-handed parallel β-helix domain, which is the core of the catalytic function of the depolymerase and is present in all the identified bacteriophage tail fibres or spike proteins with depolymerase activity; and the β sandwich domain, which has carbohydrate binding properties [18,19].

Due to the significant structural differences between lysins and depolymerases, conventional lysins may not possess depolymerase activity. However, for encapsulated *S. suis* bacteria, we speculate that natural bacteriophage may have evolved dual-activities antibacterial proteins that can simultaneously target CPS and peptidoglycan. The gut virome represents an extensive reservoir of bacteriophage-encoded proteins, many of which remain unexplored as potential antimicrobial agents [20]. In this study, we identified a bacteriophage-derived lysin, Lys33795, with dual lytic and depolymerase activities against *S. suis* from the porcine intestinal virome. Lys33795 targets both peptidoglycan and capsule through a unique multimeric architecture: an N-terminal GH25 muramidase domain and a CBD that unexpectedly retains capsule degradation activity. This dual-activity bacteriophage-derived protein effectively degrades the capsule shield, disrupts preformed biofilms, and eliminates embedded *S. suis* cells, representing a previously unrecognized class of antibiofilm agents for combating persistent infections.

## Results

### Screening and identification of candidate antibacterial proteins with dual activity of lysis and depolymerization against *S. suis*

Based on the piglet gut virome data obtained from our previous sequencing analysis (accession number CRA006589), a total of 10,166 putative lysins were identified among the 80,567 viral proteins identified, of which 280 were annotated as lysins and the remaining 9,886 were annotated as virus-associated lysins (VALs) (Fig 1A). Among these VALs, 31 were predicted to be *S. suis* lysins. These 31 proteins were subsequently analysed using a depolymerase prediction tool, and 17 candidate proteins were screened. Because bacteriophage depolymerization functional proteins typically rely on their polymeric structures to exert their activity in depolymerizing bacterial extracellular polysaccharides, we predicted the three-dimensional structures of these 17 candidate proteins and ultimately screened 11 proteins through ipTM scoring, which may exist in polymeric forms. An analysis of the structural domains revealed that among these 11 candidate proteins, 7 contain the GH25_muramidase domain, 3 encode the CHAP and amidase or MurNAc LAA catalytic domains, and 1 protein encodes the Caud_mail-N and FlgJ domains. The known binding domains include CW_7, LysM, and SH3 (Fig 1B). We validated the antibacterial activity of five selected hypothesized dual-active proteins against *S. suis* by performing experiments and evaluated the accuracy of the screening method. After three rounds of expression, the molecular weights of the three successfully expressed proteins were approximately 33 kDa (Lys33795), 33 kDa (Lys8387), and 41 kDa (Lys48548) (S1A Fig). These three proteins formed neat-edged spots on solid plates coated with *S. suis* (S1B Fig). Interestingly, as the cultivation time increased, a gradually expanding halo formed around the plaque (S1B Fig), which is similar to the plaque phenomenon produced by depolymerase on the plate [21].

**Fig 1:**
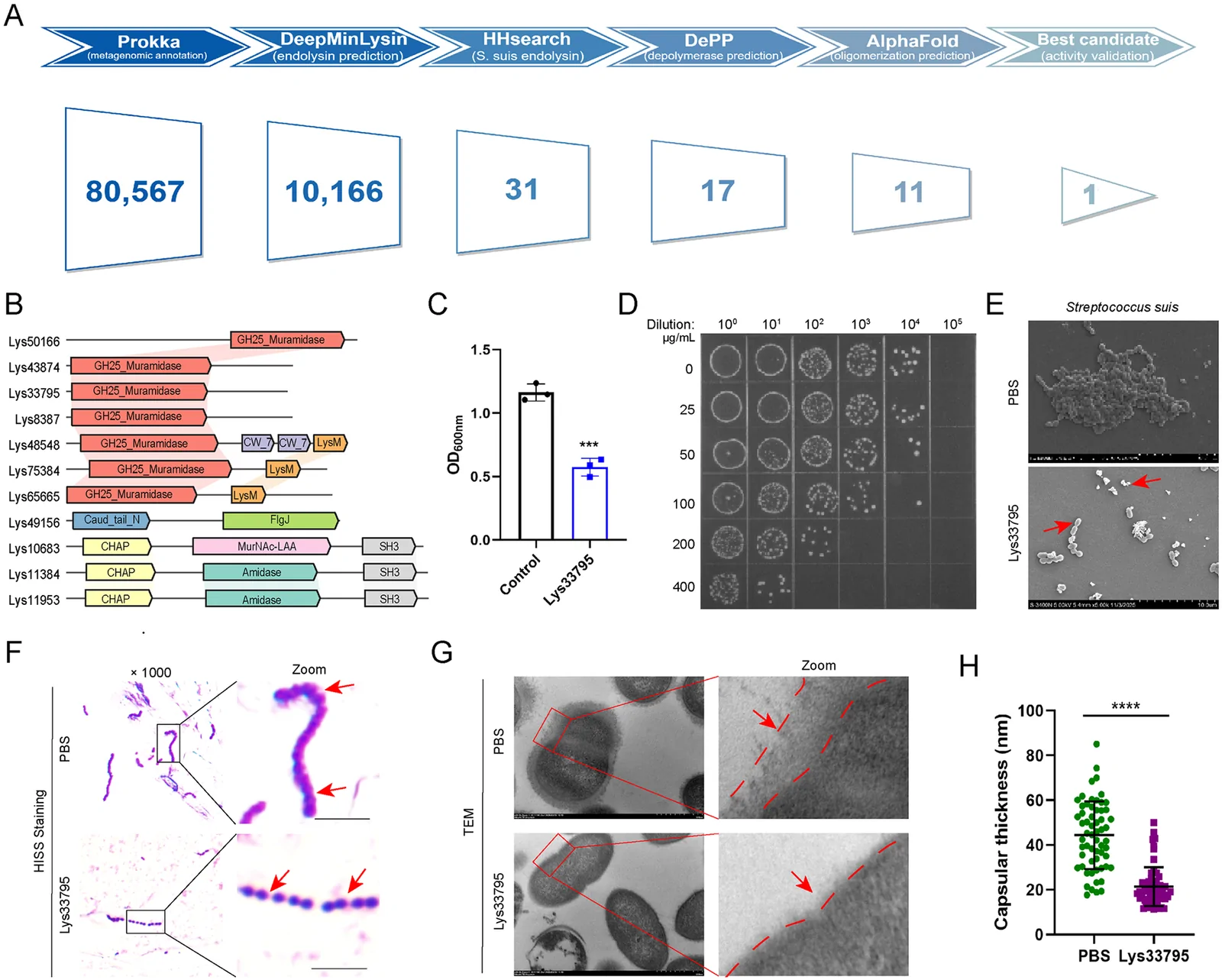
Screening and identification of antibacterial proteins with dual lytic and capsule degradation activities against *S. suis.* **(A)** The process used to screen bacteriophage antibacterial proteins with both depolymerizing and lytic activities against *S. suis.* The process includes six steps: identification of genes in the viral genome, prediction of lysin genes, prediction of *S. suis*-specific lysin genes, prediction of depolymerase genes, prediction of protein oligomers, and validation of the activity of the candidate proteins. **(B)** Visualization of the structural domains of the 11 predicted candidate proteins. **(C)** The hydrolysis activity of Lys33795 towards peptidoglycan extracted from *S. suis* SC19. Three biologically independent experiments were performed, and the data are presented as the means ± SDs; n = 3. Student’s t tests were performed. *** *P* < 0.001. **(D)** Survival of *S. suis* SC19 cultured on BHI agar dishes after treated by Lys33795 at 37 °C for 1 **h. (E)** SEM images of *S. suis* treated with Lys33795. *S. suis* treated with PBS was used as the control. Scale bar = 10 µm. **(F)** Capsular images by HISS staining (capsule: blue purple, cells: dark blue). The red arrow indicates the capsule (scale bars, 2 µm). **(G)** TEM images of *S. suis* cells treated with Lys33795 and *S. suis* cells treated with PBS was used as the control (scale bar, 500 nm. The red arrow indicates the capsule.). **(H)** Quantification of the thickness of the capsule (n = 60 cells in each group, data from three biologically independent experiments) in *S. suis* cells treated with PBS or Lys33795. Three biologically independent experiments were performed, and the data are presented as the means ± SDs. Student’s t tests were performed. **** *P* < 0.0001.

### Lys33795 has dual activities of lytic and capsule-degrading

In this study, we continued to conduct in-depth research on Lys33795. Lys33795 could hydrolyse the *S. suis* peptidoglycan and reduce the number of *S. suis* SC19 by 2 log at a concentration of 200 µg/mL (Fig 1C and 1D). Scanning electron microscopy (SEM) observations showed that the PBS-treated *S. suis* cells appears as chains, with a smooth and intact surface and a thick capsule membrane on the cell surface (Fig 1E). After treatment with Lys33795, partial bacterial cells were broken, the surface capsule of residual individual bacteria was thinner and the cell boundaries becoming more distinct compared with untreated cells (Fig 1E). In addition, the HISS staining results showed that the capsule structure of *S. suis* was significantly reduced after treatment with Lys33795 (Fig 1F). In addition, transmission electron microscopy (TEM) observation showed that compared with the PBS treated group of *S. suis* cells, the capsule thickness on the outer side of the cell wall of *S. suis* treated with Lys33795 was significantly reduced (*P* < 0.0001) (Fig 1G and 1H). Lys33795 can maintain antibacterial activity in the pH range of 2–10 and in the temperature range of 4–50 °C (S2A and S2B Fig). High concentration of EDTA (250 mM) and NaCl (750 mM) did not significantly affect the antibacterial activity of Lys33795 (S2C and S2D Fig). Importantly, Lys33795 showed a broad antibacterial spectrum against *S. suis*, reaching 75% (27/36). The antibacterial spectrum covered all serotypes detected in this study (including types 2, 3, 7, 9, 12, and 27) and Lys33795 could generate plaque and halo on all of these *S. suis* plates (S2E Fig and S1 Table). All these results indicate the excellent properties of Lys33795 as a novel antimicrobial agent against *S. suis*.

### The expression of the Lys33795 gene produces two stable peptides

PSI-BLAST (Position-Specific Iterated BLAST) showed that Lys33795 contains an N-terminal GH25_muramidase domain (residues 5–198) and a C-terminal CBD (residues 217–291). Notably, when an SDS‒PAGE analysis was performed on the purified Lys33795 protein, in addition to the 33 kDa band generated by the full-length Lys33795, we also discovered a copurified stable peptide with a molecular weight of approximately 9 kDa (Fig 2A). Size exclusion chromatography (Sec) was further performed on Lys33795 to confirm this result. Lys33795 CBD (9 kDa) and full-length Lys33795 (33 kDa) were co-eluted at 78 mL, with an estimated molecular weight of approximately 60 kDa (Fig 2B and 2C), which is higher than the theoretical molecular weight of Lys33795. These results suggest that excess CBD fragments may form polymeric complexes with full-length Lys33795.

**Fig 2:**
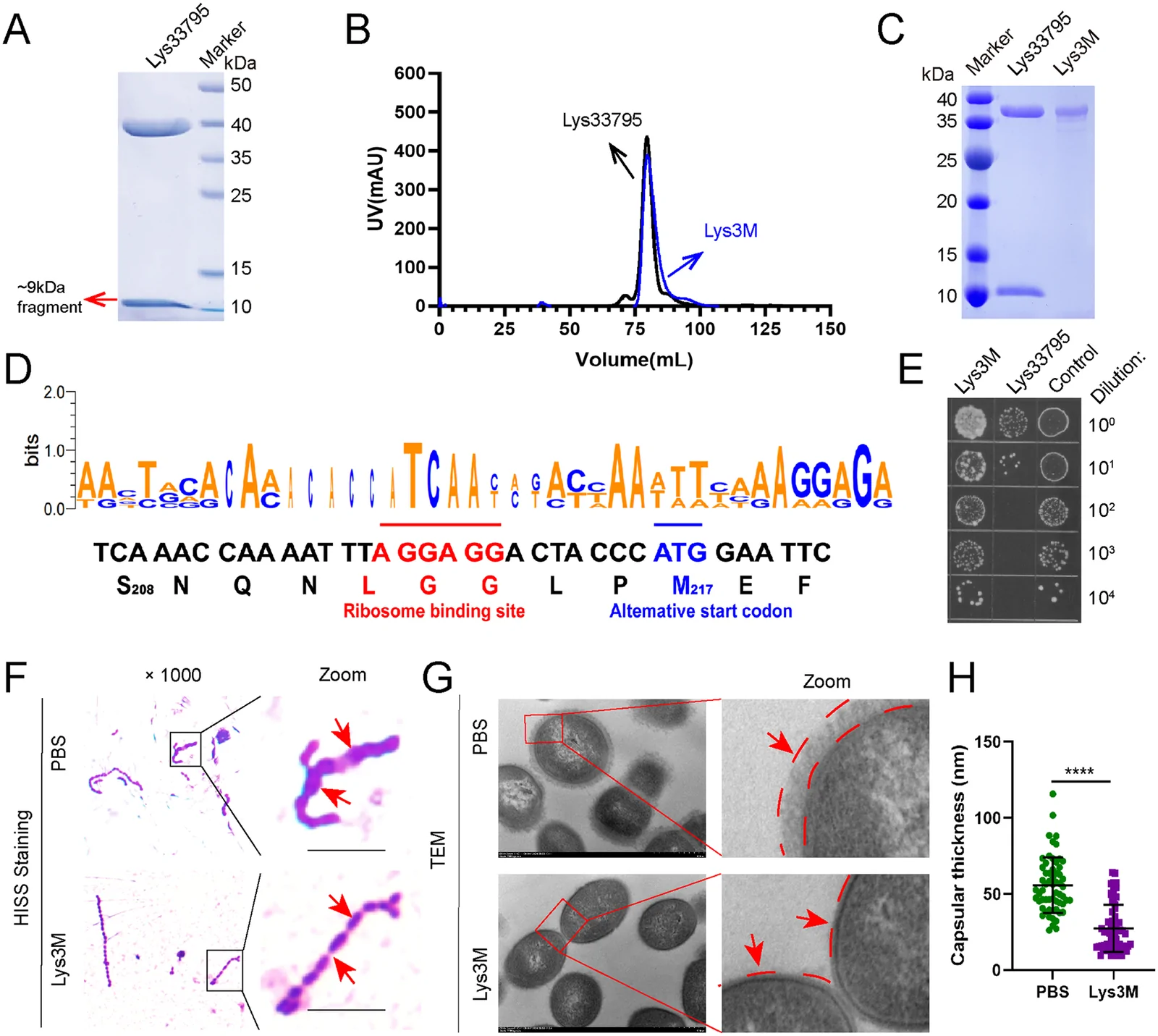
The transcription and translation of the Lys33795 gene generate two stable protein components. **(A)** SDS‒PAGE analysis of the purified of Lys33795. **(B)** Size-exclusion chromatography (GE Healthcare, Superdex 200 16/600 GL, buffer: 20 mM Tris-HCl, pH 7.5, 150 mM NaCl) of Lys33795 and Lys3M. Lys33795 and Lys3M was eluted at 78 mL. **(C)** SDS‒PAGE analGysis of Lys33795 and Lys3M. **(D)** Sequence logo. It was created with Weblogo (https://weblogo.threeplusone.com) and shown the linker region of the Lys33795 gene with nucleotides numbered according to its nucleotide sequence and based on a sequence alignment of Lys33795-related lysins. The internal ribosomal binding site sequence is underlined with a red line, and the start codon is underlined with a blue line. **(E)** Survival of *S. suis* SC19 cultured on BHI agar dishes after treated with Lys33795 (400 μg/mL) or Lys3M (400 μg/mL) at 37 °C for 1 **h. (F)** Capsular images by HISS staining (capsule: blue purple, cells: dark blue). The red arrow indicates the capsule (scale bars, 2 µm). **(G)** TEM images of *S. suis* cells treated with Lys3M. *S. suis* cells treated with PBS was used as the control (scale bar, 500 nm. The red arrow indicates the capsule.). **(H)** Quantification of the thickness of the capsule (n = 60 cells in each group, data from three biologically independent experiments) in *S. suis* cells treated with PBS or Lys3M. The data are presented as the means ± SDs. Student’s t tests were performed. **** *P* < 0.0001.

Therefore, how is the additional CBD fragment generated? We speculate that the M_217_ site may serve as an alternative starting codon site within the translation framework. Coincidentally, after the amino acid and nucleotide sequences of Lys33795 were further analysed, a hypothetical ribosome binding site was identified upstream of the M_217_ site (Fig 2D). Simultaneous mutations were generated at the M_217_ site and ribosome binding site to determine the relationship between these sequences and the production of the truncated CBD, and the double-mutant protein was named Lys3M. After Lys3M was analyzed by Sec, it was consistent with the wild-type Lys33795, and Lys3M was also eluted at near 78 mL (Fig 2B). But unlike wild type Lys33795, Lys3M was transformed into a single band with a molecular weight of approximately 33 kDa, which was consistent with that of the full-length Lys33795, while the 9 kDa product band was missing (Fig 2C). In addition, compared with Lys33795, the Lys3M mutant did not reduce the number of viable cells after treatment with *S. suis* (Fig 2E), but still significantly reduced the capsule thickness of *S. suis* (Fig 2F, 2G and 2H). These results clearly confirm that Lys3M lost its lytic activity against *S. suis* and retained only its degradation activity against *S. suis* capsules.

### Structural analysis of the Lys33795

Based on AlphaFold3 prediction, the N-terminal and C-terminal domains of Lys33795 were predicted and analysed to better understand the structural basis of its lytic and capsule degradation activities. The N-terminal GH25_muramidase domain of Lys33795 folds into a typical spherical domain, containing 6 alpha helices and 9 beta chains (Fig 3A). According to the Dali search of the PDB database, the most homologous structure is the GH25_muramidase domain of the bacteriophage-derived lysin Cpl-1 (PDB: 2J8G) from *Streptococcus pneumoniae* (Fig 3B). The sequence homology between the N-terminal GH25_muramidase domain of Lys33795 and the GH25_muramidase domain of Cpl-1 was 51%. The structural alignment between the Lys33795 and Cpl-1 domains showed that 6 alpha helices and 9 beta chains were well aligned with each other, with an average root mean square deviation (RMSD) of 0.335 Å (Fig 3C).

**Fig 3:**
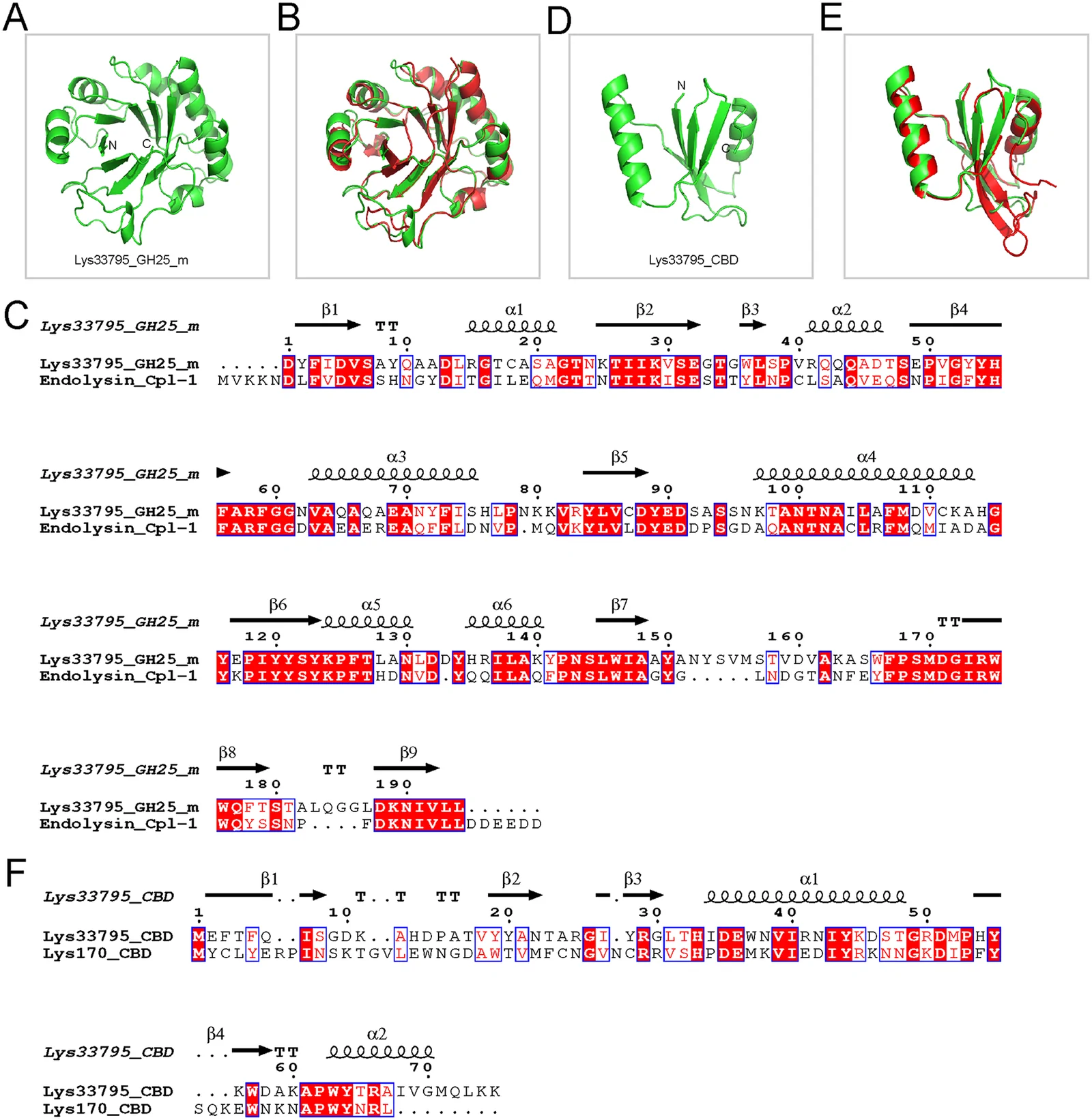
Functional domain analysis of Lys33795. **(A)** The structure of the GH25_muramidase domain of Lys33795. **(B)** Superposition of the GH25_muramidase domains of Lys33795 and Cpl-1. **(C)** Structure-based sequence alignment of GH25_muramidase (GH25_m) domains between Lys33795 and Cpl-1. Strictly conserved residues are boxed in white on a red background, and highly conserved residues are boxed in red on a white background. At the top of the sequences, the secondary structural elements of Lys33795 GH25_muramidase are shown, and every 10 residues are indicated with a dot (.) shown above the sequences. **(D)** The structure of the CBD of Lys33795. **(E)** Superposition of the CBD of Lys33795 and Lys170. **(F)** Structure-based sequence alignment of Lys33795 and Lys170 CBD. Strictly conserved residues are boxed in white on a red background, and highly conserved residues are boxed in red on a white background. At the top of the sequences, the secondary structure elements of Lys33795 CBD are shown, and every 10 residues are indicated with a dot (.) shown above the sequences. The sequence alignment was generated using ClustalW (https://www.genome.jp/tools-bin/clustalw), and the figure was generated using ESpript 3 (http://espript.ibcp.fr/ESPript/ESPript/).

The C-terminal CBD of Lys33795 consists of 2 alpha helices and 4 beta sheets (Fig 3D). According to the Dali search of the PDB database, the most closely related structure is the CBD of Lys170 (PDB: 7D55), a bacteriophage-derived lysin from *Enterococcus* (Fig 3E). The sequence homology between the C-terminal CBD of Lys33795 and the CBD domain of Lys170 was 27.7%. The structural alignment between the CBD domains of Lys170 and Lys33795 showed that the two alpha helices and four beta sheets were well aligned, with an average RMSD of 1.358 Å (Fig 3F). Coincidentally, Lys170 is also a two-component, multimeric lysin encoded by a single gene, and its structure consists of four almost identical Lys170 CBDs [22]. Based on the molecular weight determined by Sec (approximately 60 kDa), we speculate that in addition to full-length Lys33795, there may be an uncertain amount of CBD that forms a polymer with the full-length Lys33795.

### The key residues of Lys33795 responsible for lytic and depolymerizing activities

Further, we identified potential key amino acid sites related to activity in the GH25_muramidase domain and in the CBD of Lys33795 (Fig 4A and 4B) and generated single point mutants. The bactericidal activity test results showed that when residues I243, W273, W279, R282, D9, E94, or Y125 was mutated, the bactericidal activity of Lys33795 decreased. Especially after residue I243 was mutated, Lys33795's bactericidal activity was almost lost, indicating that residue I243 was the key active site in Lys33795's bactericidal activity (Fig 4C). In addition, the capsule degradation activity of Lys33795 mutant towards *S. suis* was observed by HISS capsule staining method. The results showed that after the mutation of residues I243, Y244, D251, D9 or Y125, Lys33795 lost its capsule degradation activity towards *S. suis*, indicating that residues I243, Y244, D251, D9 and Y125 were the key active sites for capsule degradation activity of Lys33795 (Fig 4D, S3 Fig).

**Fig 4:**
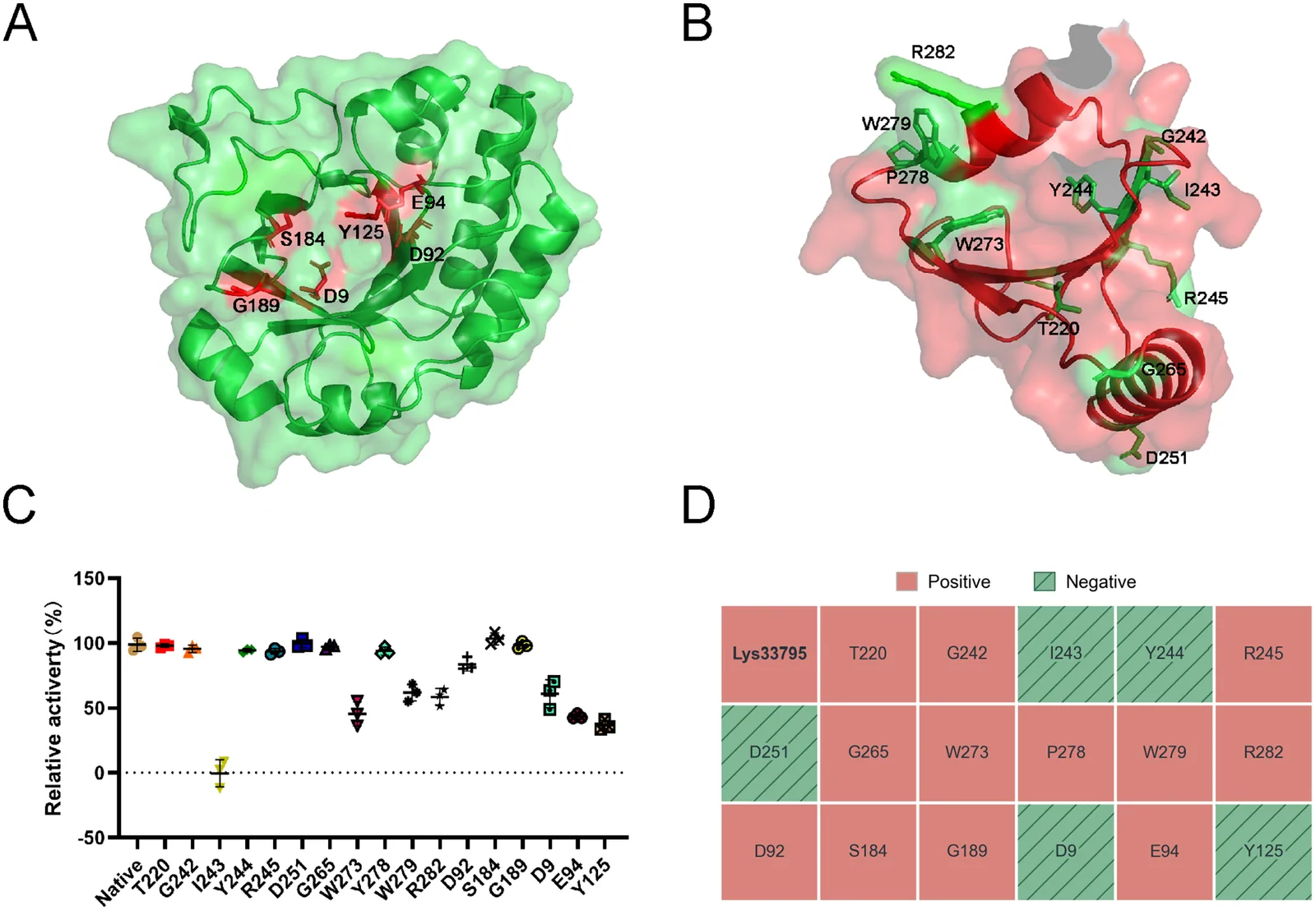
Determination of key amino acid residues in Lys33795 responsible for the lytic and capsule degradation activities. Based on the three-dimensional structural model alignment of N-terminal GH25_muramidase and results of the amino acid sequence comparison of the C-terminal CBD of Lys33795, the potential active sites of the GH25_muramidas domain **(A)** and CBD **(B)** were determined based on the conserved residues. **(C)** The bactericidal activity of Lys33795 mutants against *S. suis* SC19 and the relative lytic activity was measured. A decrease in bacterial count of 2 Log10 was considered 100% activity. Three biologically independent experiments were performed, and the data are presented as the means ± SDs; n = 3. **(D)** Statistical analysis of the degradation activity of Lys33795 mutant on the capsule of *S. suis*. Positive indicates that the mutant retained the activity of degrading the cell capsules. Negative indicates that the mutant lost its ability to degrade the cell capsules.

### Functional analysis of the truncated domain of Lys33795

We evaluated the effects of each domain of Lys33795 on lytic and depolymerization activities by constructing and expressing the individual GH25_muramidase domain and CBD respectively. (Fig 5A). Sec analysis showed that the molecular weight of the GH25_muramidase truncated fragment was approximately 22 kDa, which is consistent with the theoretical size (S4A and S4B Fig). Interestingly, GH25_muramidase, a peptidoglycan hydrolase, not only maintained binding and lytic activities against *S. suis* (Fig 5B, 5C, and 5D) but also exhibited degradation activity against *S. suis* capsule (Fig 5E, 5F and 5G).

**Fig 5:**
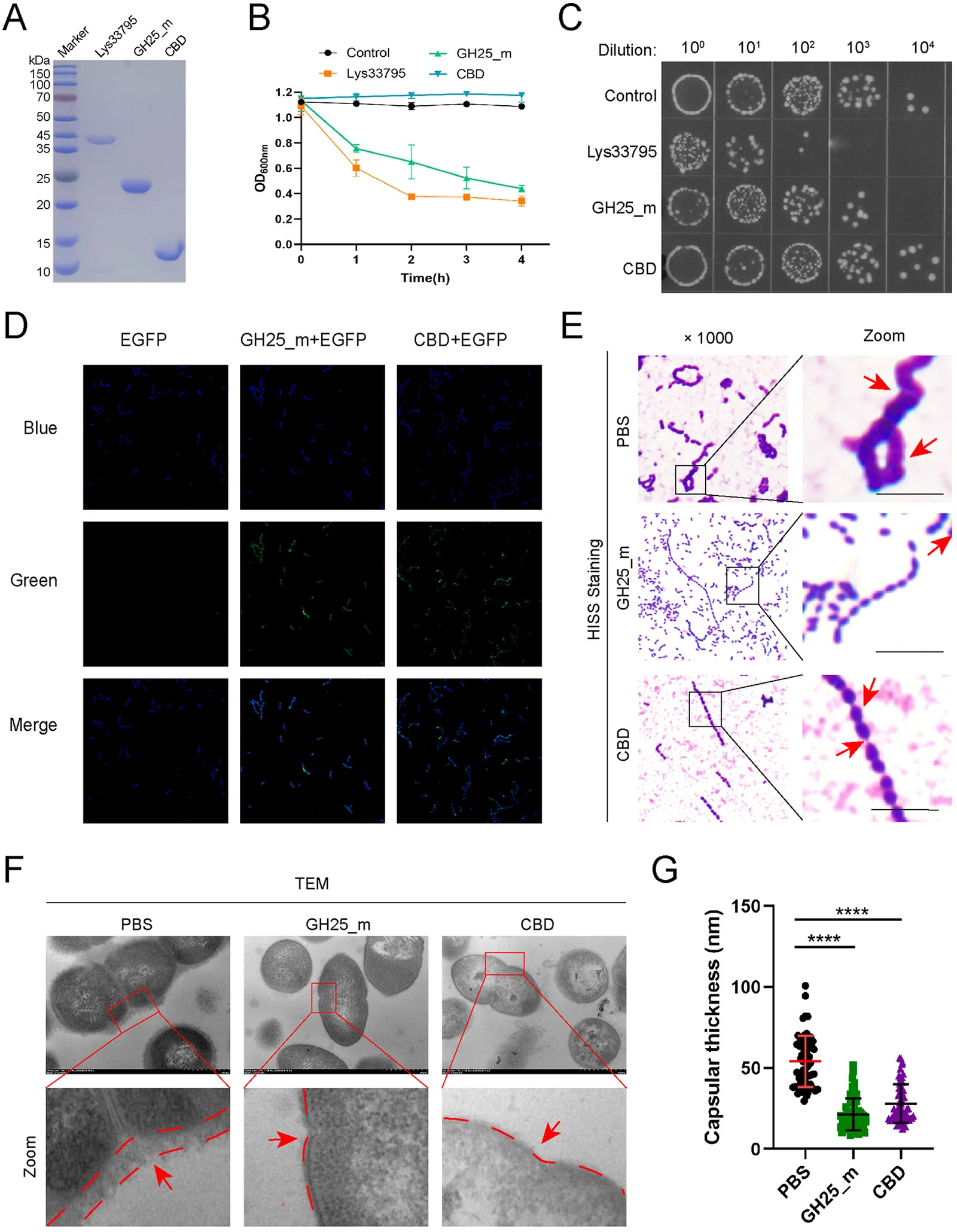
The activity of the GH25_muramidase domain and the CBD fragment. **(A)** SDS‒PAGE analysis of the purified of the truncated GH25_muramidase (GH25_m) and CBD. **(B)** The hydrolysis activity of the Lys33795, GH25_muramidase truncated fragment and CBD truncated fragment alone towards the peptidoglycan of *S. suis* SC19. Three biologically independent experiments were performed, and the data are presented as the means ± SDs; n = 3. **(C)** Survival of *S. suis* SC19 on BHI agar dishes after treated with Lys33795 (200 μg/mL), GH25_muramidase (GH25_m) (200 μg/mL) or CBD (200 μg/mL) at 37 °C. **(D)** Binding activity of truncated fragments of Lys33795. Blue, localization at 405 nm and emitted by Hoechst No. 33342; green, localization at 488 nm and emitted by EGFP; and bright, normal light. The merging of blue and green indicates the colocalization of each recombinant protein with *S. suis* SC19. **(E)** Capsular images by HISS staining (capsule: blue purple, cells: dark blue). The red arrow indicates the capsule (scale bars, 2 µm). **(F)** TEM images of *S. suis* cells treated with GH25_m or CBD. *S. suis* cells treated with PBS was used as the control (scale bar, 500 nm. The red arrow indicates the capsule.). **(G)** Quantification of the thickness of the capsule (n = 60 cells in each group, data from three biologically independent experiments) in *S. suis* cells treated with PBS or GH25_m or CBD. The data are presented as the means ± SDs. Student’s t tests were performed. **** *P* < 0.0001.

In addition, Sec analysis revealed that the molecular weight of Lys33795 CBD was approximately 18 kDa (S4C and S4D Fig), which is twice the molecular weight of monomeric CBD, indicating that CBD forms a stable dimer in solution. We further predicted the structure of the CBD dimer using AlphaFold3, and the interface-predicted TM score (ipTM) and predicted TM (PTM) values of the model were 0.77 and 0.83, respectively, indicating the high reliability of the predicted model (S5 Fig). Surprisingly, Lys33795 CBD exhibited depolymerizing activity against *S. suis* CPS in addition to maintaining its cell wall binding function (Fig 5E, 5F and 5G) but did not show obvious lytic activity against *S. suis* (Fig 5C).

Based on the aforementioned results, GH25_muramidase domain exhibits cell wall binding activity, capsule degradation activity activity, and bactericidal activity simultaneously, while CBD exhibits both cell wall binding activity and capsule degradation activity.

### The anti-biofilm ability of Lys33795 *in vitro*

We further investigated whether the dual activities of Lys33795 are more advantageous for its anti-biofilm effect. We compared the biological activity of WT Lys33795 and its single activity mutants D251 (with only lytic activity) and Lys3M (with only depolymerization activity) against the bacterial biofilms. The results of crystal violet staining showed that 200 μg/mL Lys33795 could effectively prevent and eliminate the biofilm biomass generated by *S. suis* and reduce the number of viable cells in the biofilm (Fig 6A and 6B). In contrast, the mutant D251, which only has lytic activity, has a similar ability to prevent the production of biofilm biomass as Lys33795. However, the Lys3M mutant, which only has depolymerizing activity, effectively prevented biofilm biomass formation but did not effectively decrease the number of viable cells in the biofilms (Fig 6B).

**Fig 6:**
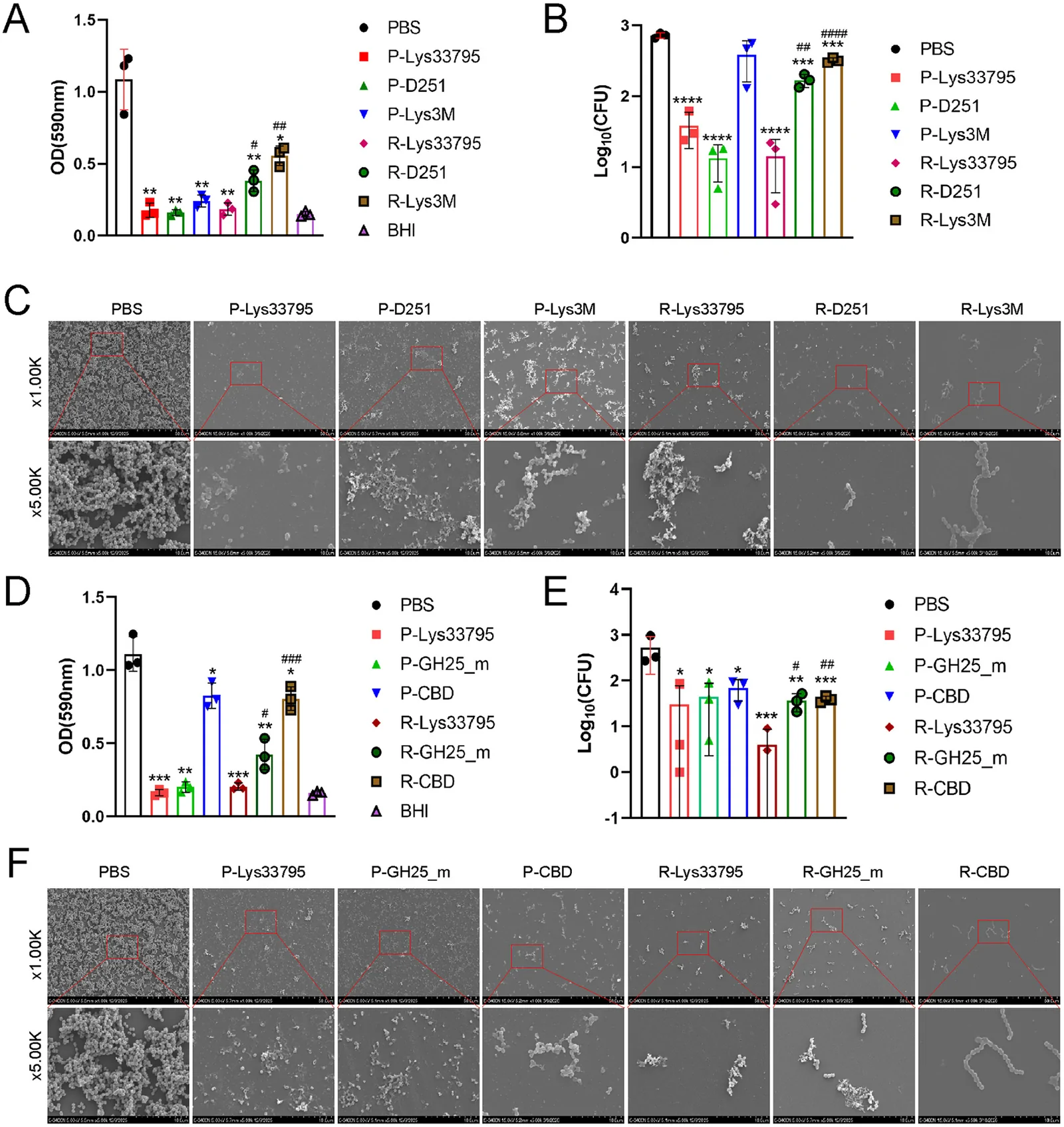
The antibiofilm activity of Lys33795, the single active mutants of Lys33795 and the truncated domain fragments *in vitro.* **(A)** Crystal violet staining was used to determine the effects of Lys33795 and its single active mutants on preventing and clearing *S. suis* biofilms. The prevention group consists of P-Lys33795, P-D251, and P-Lys3M. The clearing group consists of R-Lys33795, R-D251, and R-Lys3M. PBS treatment was used as the negative control, while BHI medium was used as the blank control. Three biologically separate experiments were performed, and the data are presented as the means ± SDs; n = 3. **(B)** The preventive and clearance effects of Lys33795 and its single active mutants on viable cells in *S. suis* biofilms. PBS treatment was used as a negative control. Three biologically separate experiments were performed, and the data are presented as the means ± SDs; n = 3. **(C)** SEM micrographs of *S. suis* biofilms treated with Lys33795 and its single active mutants *in vitro*; biofilms were treated with PBS as a control. **(D)** Crystal violet staining was used to determine the preventive and clearance effects of the truncated domains of Lys33795 on the formation of *S. suis* biofilms. The prevention group consists of P-Lys33795, P-GH25_m, and P-CBD. The clearance group consists of R-Lys33795, R- GH25_m and R- CBD. PBS treatment was used as the negative control, while BHI medium was used as the blank control. Three biologically separate experiments were performed, and the data are presented as the means ± SDs; n = 3. **(E)** The preventive and clearance effects of Lys33795 and its truncated domains on viable cells in *S. suis* biofilms. PBS treatment was used as a negative control. Three biologically separate experiments were performed, and the data are presented as the means ± SDs; n = 3. **(F)** SEM micrographs of *S. suis* biofilms treated with Lys33795 and its truncated fragments of domains *in vitro*; biofilms were treated with PBS as a control. Data were analyzed using a student’s t test or a one-way ANOVA and Tukey analysis, followed by least significant difference analysis. * *P* < 0.05, ** *P* < 0.01, *** *P* < 0.001, **** *P* < 0.0001, ^#^ *P* < 0.05, ^##^ *P* < 0.01, ^###^ *P* < 0.001, and ^####^ *P* < 0.0001. * represents a comparison with the control group, and ^#^ represents a comparison with the Lys33795-treated group.

In addition, both D251 and Lys3M effectively removed mature biofilms biomass and the number of viable cells in the biofilm, but the effects were not as good as that of Lys33795 (Fig 6A and 6B). D251 can remove about 64.88% of the total biofilm biomass, while Lys3M can remove about 48.75% of the total biofilm biomass (Fig 6A). For the number of viable cells in the biofilm, D251 can remove about 76.69% of the viable cells, while Lys3M can remove about 55.47% of the viable cells (Fig 6B). The above data indicates that the contribution of a single lytic activity to the biofilm is greater than that of a single depolymerization activity. The SEM observation results showed that, after 72 h of cultivation, *S. suis* formed a dense biofilm on the slides, which contained viable cells (Fig 6C). In contrast, the Lys33795 treatment group showed almost no biofilm or viable cells, and the effect was better than that of a single active mutant D251 and Lys3M (Fig 6C). These results indicate that Lys33795 exerts efficient anti-biofilm activity through its dual activities.

We further investigated whether the anti-biofilm activity of Lys33795 depends on the integrity of the combination of GH25_muramidase domain and CBD by comparing the anti-biofilm activity of Lys33795 and with the single GH25_muramidase domain and the single CBD, respectively. The results of crystal violet staining showed that the single GH25_muramidase domain and the single CBD had preventive and clearance effects on *S. suis* biofilms, and GH25_muramidase truncated fragment had stronger anti-biofilm activity than CBD, the effect of Lys33795 treatment was superior to that of any one single domain (Fig 6D). Lys33795, the GH25_muramidase domain and the CBD, significantly reduced the number of viable cells in the biofilm. The Lys33795 treatment was superior to treatment with its truncated form (Fig 6D and 6E). The SEM results further confirm this point. Compared with the PBS-treated group, the Lys33795-treated group showed almost no biofilm formation and no viable cells (Fig 6F). The biofilm disappeared in the GH25_muramidase-treated group, almost no viable bacteria were observed in the prevention group, and only a small number of viable cells were observed in the clearance group. The integrity of the biofilm in the truncated CBD prevention group was damaged, and the number of dead cells inside the membrane increased, whereas the biofilm was almost invisible and contained only a small number of live bacteria in the clearance group (Fig 6F). These results indicate that Lys33795 efficiently exert anti-biofilm activity depending on its structural integrity.

### Lys33795 exhibits excellent biofilm clearance ability *in vivo*

We implanted titanium plates with mature *S. suis* biofilms under the skin of mice to construct a biofilm model *in vivo*. Ampicillin and PBS were used as positive and negative controls, respectively, to evaluate the antibiofilm activities of Lys33795, its truncated protein GH25_muramidase, and CBD *in vivo* (Fig 7A). The cytotoxicity test results showed that at a concentration of 1 mg/mL, no significant cytotoxicity was observed in RAW264.7 and Caco-2 cells line (Fig 7B and 7C). In addition, the levels of endotoxins in the protein solution used for *in vivo* treatment are approximately 0.445 EU/mL (Lys33795), 0.452 EU/mL (GH25_muramidase), and 0.433 EU/mL (CBD) (Fig 7D), all below the recognized threshold for intraperitoneal or subcutaneous administration in mice (< 1 EU/mg) [23]. In *in vivo* experiments, compared with the PBS treatment group, both Lys33795, GH25_muramidase, and CBD could effectively reduce the number of viable cells on the peptide sheet, and combined treatment with antibiotics could further reduce the number of viable cells, producing an enhanced effect (Fig 7E). The SEM and laser scanning microscope (CLSM) results were consistent (Fig 7F and 7G), and a dense and thick biofilm was attached to the titanium plate in the PBS treatment group, which contained viable *S. suis*. Antibiotic treatment could disrupt and remove some biofilms, and residual biofilms contained viable *S. suis*. Compared with antibiotics, Lys33795, GH25_muramidase, and CBD could also significantly remove biofilms on titanium plates. When combined with ampicillin, the biofilm clearance effect of the combination therapy was enhanced.

**Fig 7:**
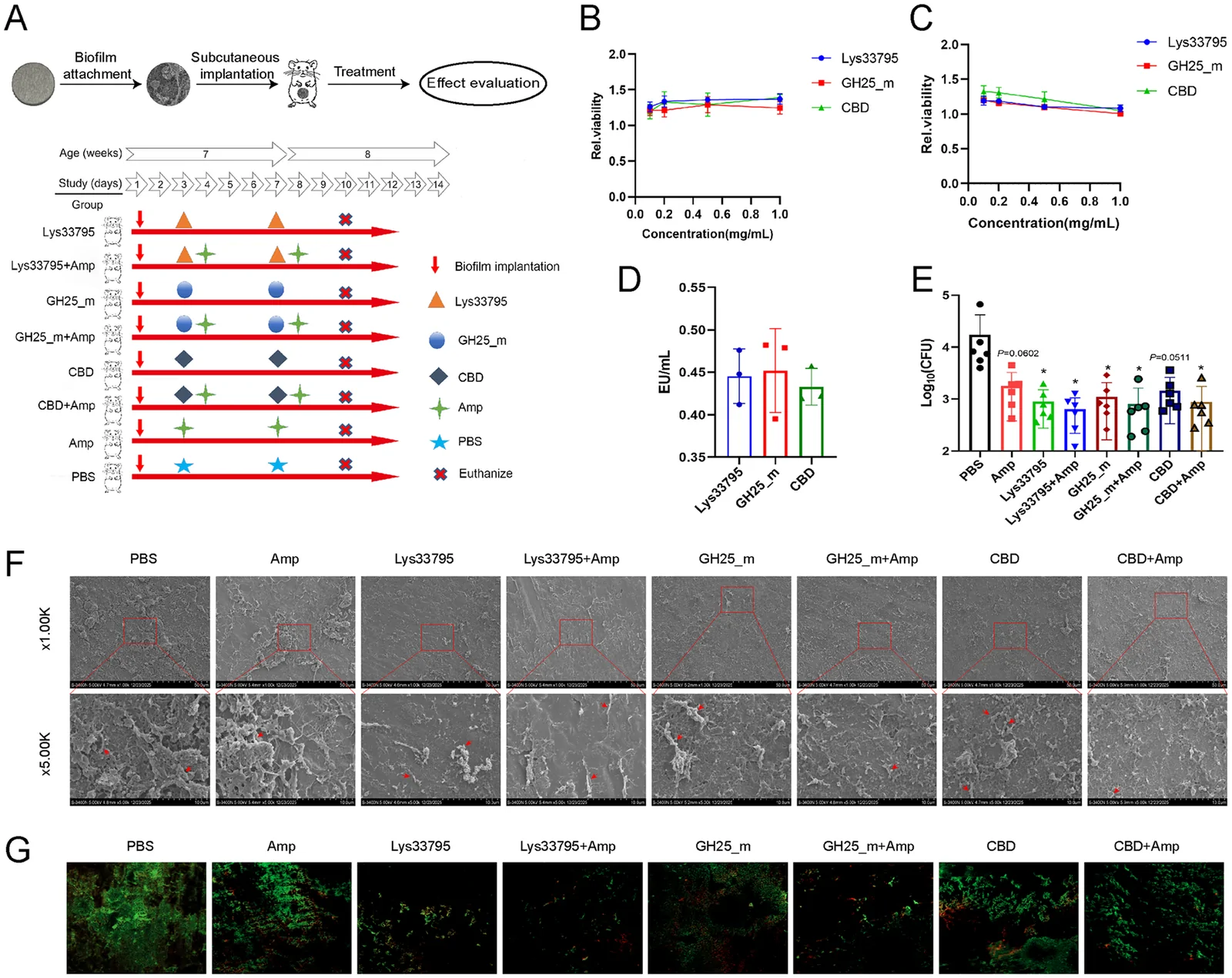
The antibiofilm activity of Lys33795 and its truncated domains *in vivo.* **(A)** A mouse model of biofilm infection caused by *S. suis* was established, and an experimental protocol for its treatment was developed. **(B and C)** The toxicity of Lys33795, GH25_muramidase truncated fragment, and CBD truncated fragment towards RAW264.7 **(B)** and Caco-2 **(C)** cells. The data are presented as the means ± SDs; n = 6. **(D)** Determination of endotoxin content in Lys33795, GH25_muramidase, and CBD solutions used for *in vivo* treatment. Three biologically separate experiments were performed, and the data are presented as the means ± SDs; n = 3. **(E)** Counting of viable cell colonies on titanium plates. **(F)** SEM micrographs of *S. suis* biofilms treated with antibiotic, Lys33795, GH25_muramidase truncated fragment, and CBD truncated fragment *in vivo*, and biofilms treated with PBS as a control. The data are presented as the means ± SDs; n = 6. Data were analyzed using a one-way ANOVA followed by Tukey analysis. * *P* < 0.05, * represents a comparison with the control group. **(G)** CLSM images of *S. suis* biofilms treated with antibiotic, Lys33795, GH25_muramidase truncated fragment, and CBD truncated fragment *in vivo*, and *S. suis* treated with PBS as a control.

## Discussion

In this study, Lys33795, a bacteriophage-derived antimicrobial protein with both lytic and capsule degradation activities, exhibits noticeable difference from previously characterized *S. suis* lysins. The observation that Lys33795 simultaneously degrades both peptidoglycan and capsule challenges the conventional functional view of bacteriophage lysins [24]. The fact that a single protein, without genetic fusion or formulation with a separate depolymerase, accomplishes two mechanistically distinct antibacterial functions underscores the potential of mining the gut virome for previously unrecognized multifunctional proteins. Importantly, this study provides a universal pipeline for the discovery of dual-activity antibacterial proteins by screening the capsule degradation activity of bacteriophage-derived lysins through structure-oriented prediction of multiple activities. Given that the vast majority of virus sequences in the metagenomic dataset have not been functionally labelled, the virome represents an undeveloped library of antimicrobial proteins [20,25,26].

Lys33795 comprises a GH25 muramidase domain and a CBD domain. Interestingly, Lys33795 may forms an oligomeric complex comprising one full-length protein and additional CBD fragments, and both the M_217_ site and the predicted RBS site are associated with the production of 9 kDa CBD fragments and Lys33795 lytic activity. However, SEC alone cannot determine the exact stoichiometry, and the decreased activity of Lys3M and point mutants may be partially due to changes in folding, stability, or oligomerization. Therefore, more direct or orthogonal evidence of translation products and oligomeric states is needed to conduct in-depth analysis of the oligomeric structure and mechanism of action of Lys33795. This multimeric assembly is similar to the well-characterized streptococcal bacteriophag lysin PlyC, which is composed of one PlyCA catalytic subunit (harbouring two synergistically acting catalytic domains, a GH25 muramidase domain and a CHAP domain) and eight PlyCB binding subunits that assemble into an octameric ring [27]. Although PlyC is encoded by two separate genes (plyCA and plyCB) transcribed from a single operon, Lys33795 is encoded by a single gene but may be restarted through translation at the internal start codon (M_217_) and the putative ribosome binding site upstream of that codon.

The presence of secondary translation products has been described for several lysins of bacteriophage [28,29], which is consistent with the generally accepted notion that bacteriophage makes very economical use of their genetic material. With respect to oligomerization, only four lysins have been reported to form active multimers: CTP1L and CD27L derived from Clostridium tyrobutyricum phages, and LysIME-EF1 and Lys170 derived from *Enterococcus faecalis* bacteriophage [30–33]. Zhou et al. reported that the use of an internal translation site may be common among lysin genes [30]. The conservation of this genetic architecture across different bacteriophage systems suggests that it may represent a widespread but underappreciated strategy for generating functional multimeric lysin complexes from compact bacteriophage genomes. However, except for the three *S. suis* bacteriophage lysins identified in this study, which have both lytic and capsule degradation activities, the others lysins have been reported to only possess lytic activity, especially those four lysins mentioned above may retain depolymerizing activity against host bacterial capsule. Thus, their functions and mechanisms of action need further investigation.

An unexpected finding in this study is that the GH25 muramidase domain of Lys33795 not only retains its classical function as a peptidoglycan hydrolase for degrading bacterial cell walls but also exhibits degradation activity towards capsule. This discovery contrasts sharply with the traditional understanding of the function of the GH25 family. According to the classification in the CAZy database, the GH25 family belongs to the lysosome type of the glycoside hydrolase family. Its defined biochemical function is to specifically hydrolyse the β-1,4-glycosidic bond between N-acetyl muramic acid (NAM) and N-acetylglucosamine (NAG) in the bacterial peptidoglycan skeleton [34]. Bacteriophage depolymerases typically appear as structural components of bacteriophage particles, mainly in forms such as tail spike proteins (TSPs). They specifically bind to CPS, extracellular polysaccharides (EPSs), or lipopolysaccharides (LPSs) on the surface of host bacteria, accurately cleaving polysaccharide recombination units and assisting bacteriophage in breaking through the bacterial extracellular polysaccharide barrier [35]. Structurally, these depolymerases typically adopt independent polymeric architectures. For example, the tail spike protein K2-2 of the *Klebsiella pneumoniae* K2 bacteriophage forms a trimeric structure and carbohydrate binding grooves between its subunit interfaces to accommodate K2 CPS substrates [36]. However, the GH25 muramidase domain in this study is only composed of the N-terminal 198 amino acids of Lys33795 and does not possess the typical multimeric architecture of tail spike proteins, nor does it contain any known characteristic domains of CPS-degrading enzymes. To date, all reported GH25 family enzymes have been reported to function in peptidoglycan hydrolysis, and no any literature has shown that the GH25 domain has degradative activity against bacterial extracellular polysaccharides (including CPS). Therefore, the GH25 domain has dual functions with both peptidoglycan hydrolysis and capsule degradation, which goes beyond the traditional functional definition of the GH25 domain and reveals unprecedented substrate spectrum expansion potential for this enzyme family. Future research can further elucidate the molecular mechanism of this unconventional function by analysing the three-dimensional structure of the GH25 domain and CPS substrate and identifying key amino acid residues involved in CPS degradation.

Another interesting finding of this study is that the isolated CBD of Lys33795 retains capsule degradation activity against *S. suis* CPS. The CBD of Lys33795 lacks sequence homology to any known depolymerase catalytic domains, but Sec analysis revealed that the CBD alone forms a dimer, suggesting that oligomerization may be essential for its depolymerase function. These observations challenge the conventional view that CBDs serve exclusively as cell wall-anchoring modules that direct the catalytic domain to its peptidoglycan substrate [12,37]. Instead, our results suggest that certain CBDs may possess intrinsic polysaccharide-binding and polysaccharide-degrading capabilities, possibly through a noncanonical mechanism involving multivalent binding-induced conformational disruption of the CPS matrix rather than classical hydrolytic cleavage. Alternatively, the CBD may recruit host-encoded or environmental factors that contribute to CPS degradation, a possibility that warrants further investigation through cocrystallization studies and glycan array analyses.

The *in vivo* anti-biofilm efficacy observed in the subcutaneous titanium plate model of mice confirms that Lys33795 has the potential to serve as a therapeutic agent for chronic device related infections. Strikingly, combination therapy with Lys33795 and ampicillin resulted in the near-complete eradication of biofilms from the titanium plates, indicating that the combination of these two antibacterial substances can produce an enhanced effect. This finding is expected, as previous studies have shown that the absence of capsule protection significantly increases the sensitivity of *S. suis* to antibiotics such as ampicillin [38]. Therefore, Lys33795 degrades the biofilm of *S. suis* and degrades the polysaccharides in the bacterial capsule, which is beneficial for antibiotic contact and the killing of *S. suis* exposed in the biofilm. In summary, Lys33795 exemplifies a novel class of multifunctional, virome-derived antimicrobial proteins that combines biofilm disruption with direct bacterial killing, providing a blueprint for developing next-generation agents to combat persistent, biofilm-mediated bacterial infections.

## Materials and methods

### Ethics statement

All the animal experiments conducted in this study were approved by the Institutional Animal Care and Use Committee (IACUC) of Jilin University, Jilin, China (no. SY202511039), and the procedures strictly followed the experimental animal care and use manual published by the U.S. National Institutes of Health.

### Animals

Female C57BL/6 mice aged 6–8 weeks were purchased from Liaoning Changsheng Experimental Animals (Liaoning, China). All the mice were housed under normal conditions for one week to adapt to their new surroundings.

### Bacterial strains and culture conditions

All *S. suis* strains used in this study were maintained as laboratory stocks. All *S. suis* strains were routinely cultured in brain heart infusion (BHI) broth (Becton, Dickinson and Company, USA) or on agar plates at 37 °C for 8–12 h.

### Strategy for screening dual-active bacteriophage antibacterial proteins

The screening process for bacteriophage antibacterial proteins with both depolymerizing and lytic activities against *S. suis* is shown in Fig 1A. Specifically, raw virome sequencing data were obtained from the Genome Sequence Archive (GSA) (https://bigd.big.ac.cn/gsub/) at the BIG Data Center, Beijing Institute of Genomics, Chinese Academy of Sciences, under accession number CRA006589. The quality control and assembly of the raw data were performed as described in previous studies [25]. The Prokka tool was used for gene predictions and functional annotations [39], and the predicted protein coding sequences (CDSs) were used for subsequent screens of functional proteins. The DeepminLysin deep learning model was used to predict bacteriophage lysins in protein sequences predicted by Prokka [40]. Sequences with predicted scores ≥ 0.5 were selected as candidate lysins. HHsearch was subsequently used to compare the candidate sequences with those in the *S. suis* lysin database to identify potential *S. suis*-specific lysins. Based on the screened potential *S. suis*-specific lysin genes, the DePP (Depolymerase Predictor) tool was used to further predict bacteriophage depolymerase genes [41]. The screening criterion was a prediction probability of ≥ 0.5, and further validation was conducted by combining the sequence length (≥ 200 aa) and analysis of conserved domains. The AlphaFold Multimer tool was used to predict the polymer structure of the selected candidate proteins [42]. The prediction parameters are set to the defaults, and the reliability of the predicted structure was evaluated by measuring the following indicators: different aggregation forms of the ipTM score were compared, and the best result was used as a reference result, and the predicted PTM score ≥ 0.5 was used as the overall structural credibility threshold. The predicted structure was aligned with that of homologous proteins in the PDB database using TM align, and the assembly form of the quaternary structure of the protein was inferred based on the ipTM score. PyMOL (https://www.pymol.org/pymol.html?) was used for a visual analysis of the predicted protein structures. By comparing these structures with the known crystal structure of the depolymerase, combined with an analysis of conserved residues and surface electrostatic potential calculations, the characteristic results were manually screened and identified. After the selected candidate genes were synthesized, they were cloned and inserted into an expression vector and transformed into competent *Escherichia coli* (*E. coli*) BL21 (DE3) cells for the induction of protein expression and purification, after which protein activity was measured.

### Correlation analysis of structural domains

The 11 candidate protein sequences of EFANCAML_10683 (Lys10683), EFANCAML_11384 (Lys11384), EFANCAML_11953 (Lys11953), EFANCAML_43874 (Lys43874), EFANCAML_48548 (Lys48548), EFANCAML_49156 (Lys49156), EFANCAML_50166 (Lys50166), EFANCAML_65665 (Lys65665), EFANCAML_75384 (Lys75384), EFANCAML_33795 (Lys33795), and EFANCAML_8387 (Lys8387) were submitted in FASTA format to the InterProScan 5 RESTful service of the EMBL-EBI Job Dispatcher framework (endpoint: https://www.ebi.ac.uk/Tools/services/rest/iprscan5) to annotate conserved domains [43]. The results were visualized using the ‘gggenomes’ 1. x R package in the R 4.4.3 environment. Each protein was represented by a horizontal reference line, and the structural domains were stacked in an arrow shape (geom_gene, body height 5 mm, arrow direction along the N-terminus → C-terminus) to the corresponding position.

### Constructs, expression and purification of proteins

The information on all primers used in this experiment is listed in S2 Table. The Lys33795, Lys8387, Lys48548, Lys49156 and Lys11384 genes sequence were synthesized by Sangon Biotechnology (Shanghai, China) and cloned and inserted into the pET-28a (+) vector. The recombinant plasmid was verified by sequencing. The recombinant protein was prepared using previously described methods with minor modifications [44]. Briefly, the recombinant plasmid was transformed into competent *E. coli* BL21 cells, which were subsequently cultured in LB supplemented with 100 μg/mL kanamycin at 37 °C until they reached the logarithmic growth phase (OD_600_ = 0.6). A final concentration of 1 mM IPTG was added, and the bacteria were incubated at 16 °C for 16 h to induce protein expression. The centrifuged and resuspended bacterial cells were sonicated, and the supernatant was collected by centrifugation (4 °C, 12,000 × *g*, 20 min). The protein in the supernatant was purified using Ni-NTA affinity chromatography. The protein was concentrated using ultrafiltration tubes, and the imidazole was removed by washing. The concentration was measured, and the concentrated protein was stored in a -80 °C ultralow temperature freezer after being frozen in liquid nitrogen. The purity and molecular weight of the protein were determined by performing sodium dodecyl sulfate–polyacrylamide gel electrophoresis (SDS–PAGE).

PSI-BLAST was used to analyse the Lys33795 protein sequence to identify the active domains of the protein. To determine the approximately 9 kDa peptide sequence generated by Lys33795 through SDS-page analysis, the corresponding gel strips were cut and sent to Beijing Liuhe Huada Gene Technology Co., Ltd. for protein mass spectrometry analysis. Expression vectors carrying the Lys33795 truncated fragments of GH25_muramidase (N-terminal domain, residues 1–199) and CBD (C-terminal domain, residues 200–291) were constructed as previously described [44]. In short, GH25_muramidase domain and CBD were amplified by polymerase chain reaction (PCR) and cloned and inserted into the pET-46b vector, the expression vectors pET-46b- GH25_muramidase and pET-46b-CBD were constructed. The correct recombinant plasmid was transformed into *E. coli* BL21 cells. In addition, fusion genes expression vectors consisting of GH25_muramidase or CBD fragments with enhanced green fluorescent protein (EGFP) were constructed as described previously [44]. As mentioned above, the correct recombinant plasmid pET-46b-GH25_m-EGFP and pET-46b-CBD-EGFP were expressed and purified. SDS‒PAGE was used to verify the purity and size of the proteins.

### Determination of bactericidal spectrum of Lys33795

A plaque assay was performed to determine the spot-clearing activity of Lys33795. Thirty-six strains of *S. suis* were cultured to the logarithmic growth stage. Afterwards, 100 μL of bacterial solution was evenly spread onto solid BHI plates. Next, 10 µL of purified Lys33795 (2 mg/mL) was dropped onto the solid BHI plates coated with *S. suis*, allowed to dry, and the plates were incubated in a 37 °C incubator for 16 h to observe and record the appearance of the spots. In addition, the antibacterial activity of Lys33795 against sensitive strains was further determined through quantitative CFU reduction experiments. Specifically, the strain was cultured to the logarithmic growth stage, washed three times with sterile PBS, and purified Lys33795 was added at a final concentration of 200 µg/mL. The control group was treated with an equal amount of sterile PBS. Incubated at 37 °C for 1 h and the CFU count of the sample was determined using plate counting method.

### Structure prediction and key amino acid action site analysis of Lys33795

The three-dimensional structure of Lys33795 was predicted using AlphaFold3 and visualized using PyMOL v.3.1.3.1. To predict active site residues, multiple sequence alignment was performed using MEGA software to identify conserved amino acid residues in the homologous protein sequences of GH25_muramidase and CBD. In addition, Foldseek webserve was used to compare the structure of GH25_muramidase of Lys33795 with the database PDB100. In the structural alignment results, the protein Cpl-1 (PDB ID 1OBA) with clear features and the highest matching rate was selected as the reference. Based on the active site residue of Cpl-1 and the ligand binding site prediction of PrankWeb server (https://prankweb.cz/), the estimated catalytic residue of Lys33795 was determined. Based on the results of the above two prediction methods, the key active sites assumed by Lys33795 were obtained. The predicted active site residues were all mutated to alanine. In addition, the Lys33795 sequence was analysed using the Promoter tool, and the methionine at position 217 and the ribosome binding site AGGAGG were simultaneously mutated (mutated to ACCAGG). The primers used to generate the mutants are listed in S2 Table. The PCR products were digested with the Dpn I enzyme for 5 h, after which all the digested products were transferred into *E. coli* DH5α adaptive cells. The predicted sites in Lys33795 were mutated to alanine to further confirm the critical amino acids. The primers used to generate the mutants are listed in S2 Table. The correct mutant plasmids were transformed into *E. coli* BL21 (DE3), and the Lys33795 mutants were expressed and purified as described above.

The *S. suis* SC19 bacterial solution was adjusted to 1 × 10^7^ CFU/mL, and each mutant was added at a final concentration of 200 μg/mL to determine the lytic activity of the mutant proteins against *S. suis*. The mixture was incubated at 37 °C for 1 h and continuously diluted at multiple ratios for colony counting. An equal amount of PBS buffer was added to the control group. The experiment was repeated three times.

### Binding activity of the truncated domains of Lys33795

*S. suis* cells in the logarithmic growth phase were treated with 20 µM Hoechst 33342 (Cat: B2261, Sigma‒Aldrich) fluorescent dye for 10 min at 37 °C and then were washed five times with PBS. The bacteria were resuspended in 100 µL of PBS and incubated with EGFP, GH25_m+EGFP and CBD+EGFP at 37 °C for 20 min to determine the binding of Lys33795 to bacteria, after which the cells were collected by centrifugation (8,000 × *g*, 4 °C, 5 min) and washed to remove the unbound proteins before being suspended in 100 µL of PBS. Cellular fluorescence was detected using a CLSM (Zeiss Axio Observer Z1/7, Germany) at wavelengths of 405 nm and 488 nm.

### Sec analysis

The purified Lys33795, GH25_muramidase truncated fragment, CBD truncated fragment, and Lys3M mutant proteins were analysed by Sec (Superdex 200 16/600 GL column, GE Healthcare, 20 mM Tris HCl, pH 7.5, 150 mM NaCl). The test was conducted at a flow rate of 0.5 millilitres per minute and 16 °C.

### Determination of lytic activity

*S. suis* SC19 was cultured to the logarithmic growth stage (OD_600_ = 0.8), and the bacterial solution was evenly spread on a plate containing solid BHI medium. Next, 10.0 µL of purified Lys33795 (1mg/mL), Lys8387 (1mg/mL) and Lys48548 (1mg/mL) was added to the solid medium dropwise. After the plate was dried, it was placed in a 37 °C incubator for 16 h for observed. In addition, the *S. suis* solution was mixed with Lys33795, GH25_muramidase truncated fragment, CBD truncated fragment or Lys3M mutant at a final concentration of 200 µg/mL respectively, and incubated at 37 °C for 1 h; PBS was added as a negative control. The samples that had been incubated for 1 h was diluted 10-fold and dropped onto a solid BHI plate. After being placed on a flat plate at 37 °C and cultured for 16 h, bacterial colonies were counted.

### The degradation activity of proteins toward *S. suis* peptidoglycan

As previously reported, *S. suis* peptidoglycan was extracted using the trichloroacetic acid (TCA) method [45]. Specifically, 15 g of *S. suis* SC19 bacterial cells cultured overnight were washed and resuspended in 100 mL of a 10% TCA solution. After a 20 min incubation in a boiling water bath, the samples were centrifuged (12,000 × *g*, 15 min), and the precipitate was collected and washed three times with distilled water. The precipitate was resuspended in trypsin phosphate buffer, shaken at 37 °C for 3 h, and then centrifuged (3,000 × *g*, 5 min) to remove the excess trypsin. The sample was centrifuged again (12,000 × *g*, 15 min), the precipitate was washed twice, ether was added for degreasing, and the sample was finally centrifuged at 12,000 × *g* for 15 min. After drying and dehydration, the peptidoglycan extract was obtained. The concentration of peptidoglycan extract was adjusted to an OD_600_ of 1.0. Lys33795 and GH25_muramidase truncated fragment, CBD truncated fragment was mixed peptidoglycan extract at final concentrations of 100 µg/mL respectively, and incubated for 2 h, after which the absorbance was measured.

### Bacterial capsule staining

Hiss staining method was used to stain the capsule of *S. suis* base on previous study [46]. Specifically, *S. suis* in logarithmic growth phase (OD_600_ = 0.8) was collected, washed twice with sterile PBS, and then added Lys33795, GH25_muramidase, CBD and Lys33795 mutants at a final concentration of 200 ug/mL, respectively. PBS was added as a negative control. The mixtures were incubated at 37 ℃ for 2 h, and stained according to the instructions provided by the Hiss staining kit (Bioss, S0223), and the images were obtained using an upright biological microscope (Olympus, BX53, Japan).

### TEM analysis the morphological changes of *S. suis*

To evaluate the morphological changes of *S. suis*, TEM observation was conducted based on previous studies with minor modified [46]. Specifically, *S. suis* in logarithmic growth phase (OD_600_ = 0.8) was collected, washed twice with sterile PBS, and then added Lys33795, GH25_muramidase, CBD and Lys3M at a final concentration of 200 ug/mL, respectively. PBS was added as a negative control. The mixtures were incubated at 37 ℃ for 2 h, fixed in 2.5% glutaraldehyde for 2 h, and then washed with PBS. Incubate fixed cells in 1% osmium tetroxide carboxylate buffer at room temperature in the dark for 1 h. The precipitated cells were dehydrated using an ethanol gradient for 20 min and then embedded in Epon-812 epoxy resin. The samples were stained into ultra-thin sections using uranyl acetate and lead citrate, and observed under a TEM (Hitachi Ltd., Japan) at an acceleration voltage of 100 kV.

### SEM analysis the morphological changes of *S. suis*

The samples used for the SEM analysis were prepared using a previously described procedure, with some modifications [20]. Briefly, *S. suis* SC19 cells treated with 200 µg/mL Lys33795 or PBS (negative control) were washed three times with sterile PBS and then fixed with 4% glutaraldehyde for 4 h. The bacterial cells were dehydrated with different concentrations of ethanol (10%, 30%, 50%, 70%, 90%, and 100%), dried overnight on coverslips, and observed using SEM (Hitachi S-3400N, Germany).

### Biological characterization of Lys33795

*S. suis* SC19 were cultured to the logarithmic growth stage (OD_600_ = 0.8), and the Lys33795 protein was suspended in PBS at different pH values (pH = 2–10) for 1 h or incubated at different temperatures (4 °C, 25 °C, 37 °C and 50 °C) for 1 h. The bacterial solution was incubated with the treated Lys33795 at a final concentration of 100 µg/mL and incubated in a 37 °C water bath for 1 h, the colony count was determined. As a control, the viable cell count was measured after treatment with PBS under the same conditions. All experiments were conducted in triplicate.

*S. suis* was resuspended in PBS buffer solutions containing different concentrations of NaCl (0, 50, 100, 150, 300, 500, and 750 mM), and Lys33795 was added at a final concentration of 200 µg/mL and incubated at 37 °C for 1 h, colonies were counted. Relative activity is reported as a percentage of the maximum bactericidal activity. All experiments were conducted in triplicate.

Ethylenediamine tetraacetic acid (EDTA) was used to evaluate the dependence of Lys33795 antibacterial activity on metal ions. Lys33795 was treated with 250 mM EDTA for 30 min, and then the EDTA was removed by ultrafiltration. The processed Lys33795 (at a final concentration of 200 µg/mL) was mixed with a bacterial suspension and incubated at 37 °C for 1 h, after which the colonies were counted. All experiments were conducted in triplicate.

### Cytotoxicity assay

A CCK-8 kit was used to detect the toxicity of Lys33795 to RAW264.7 and Caco-2 cells. Specifically, RAW264.7 cells and Caco-2 cells were seeded at a density of 1,000 cells per well in a 96-well plate, incubated at 37 °C with 5% CO_2_ for 24 h, and then incubated with different concentrations of protein (0, 100, 200, 500, or 1000 μg/ml) for another 24 h. The plate was then supplemented with fresh culture medium supplemented with a 10% CCK-8 solution and incubated at 37 °C for 2 h. The final optical density at 450 nm was recorded using a spectrophotometer, and the results are presented as the cell survival rate (%). PBS treatment was used as a control. Each group consisted of 6 replicates.

The Pierce colorimetric endotoxin quantification kit (A39552S) was used to determine the content of lipopolysaccharides (LPS) in Lys33795, GH25_muramidase, and CBD protein solutions used for *in vivo* therapy.

### Evaluation of the antibiofilm efficacy *in vitro*

The antibiofilm effects of Lys33795 and its truncated fragment GH25_muramidase, CBD, as well as the single mutant D251 with lytic activity and single mutant Lys3M with depolymerizing activity against *S. suis* were determined as previously described, with minor modifications [25]. Specifically, the *S. suis* SC197 bacterial solution in the logarithmic growth phase was diluted 100-fold with sterile liquid BHI medium and added to a sterile 96-well plate (200 µL per well) to determine the preventive effect of the proteins on *S. suis* biofilm. Subsequently, Lys33795, GH25_muramidase, CBD, D251 and Lys3M were added to the wells at a final concentration of 200 μg/mL. Six wells were established for each group, sterile PBS buffer was added as a negative control, and sterile BHI medium was used as a blank control. Three of the wells were used to measure the number of *S. suis* bacteria surviving in the biofilm, while the remaining three wells were used for the quantitative measurement of the biofilm using crystal violet staining. After the cells were incubated at 37 °C for 72 h, the unattached cells were discarded, the attached cells were gently washed three times with sterile PBS, and the cells in three wells from each group were fixed with methanol for 30 min. The methanol was discarded, the cells were thoroughly air dried, and 100 µL of 1% crystal violet was added to each well. The cells were stained at room temperature for 30 min and then were washed three times with PBS to remove the excess dye. Afterwards, 200 μL of a 33% glacial acetic acid solution was added, the mixture was incubated for 30 min, and an enzyme-linked immunosorbent assay (ELISA) reader was used to measure the OD_590_ value. In addition, 100 µL of sterile PBS was added to the remaining 3 wells in each group. After the biofilm at the bottom of the wells was fully dissolved, it was coated on a solid plate for colony counting. All experiments were conducted in triplicate. The average of the data from three holes in each experiment was taken as the final statistical data.

The *S. suis* SC197 bacterial culture in the logarithmic growth phase was diluted 100-fold with sterile BHI liquid medium and added to a sterile 96-well plate (200 µL per well). Six wells were established for each group. The plate was incubated at 37 °C for 72 h, after which any unattached cells were discarded. The plate was gently washed three times with sterile PBS, and 100 µL of Lys33795, GH25_muramidase, CBD, D251 and Lys3M at a final concentration of 200 μg/mL was added to well; sterile PBS buffer was added as a negative control, and sterile BHI medium was used as a blank control. After the cells were incubated at 37 °C for 1 h, the culture medium was discarded, and the cells were gently washed three times with sterile PBS. As mentioned above, 3 wells from each group were treated with methanol, 1% crystal violet, and a 33% glacial acetic acid solution, and the OD_590_ value was measured. In addition, 100 µL of sterile PBS per well was added to three wells, and the biofilm at the bottom of the wells was fully dissolved before being coated on a solid plate for colony counting. All experiments were conducted in triplicate. The average of the data from three holes in each experiment was taken as the final statistical data.

### Observations of biofilms under a SEM

As previously reported, SEM was used to observe the effects of Lys33795 and its truncated fragment GH25_muramidase and CBD on preventing and clearing *S. suis* biofilms [25,47]. Briefly, sterile glass coverslips were placed in the wells of a 12-well microplate. For the preventive treatment group, the *S. suis* SC197 suspension (1 × 10^6^ CFU/mL) was added to the well, followed by the addition of the protein at a final concentration of 200 μg/mL. The control group was treated with an equal amount of PBS as a negative control. The samples were incubated at 37 °C for 72 h, the coverslip was washed three times with sterile PBS, and the samples were fixed with 4% glutaraldehyde for 2 h. The samples were dehydrated sequentially with different concentrations of ethanol (10%, 20%, 50%, 70%, 90%, and 100%) for 30 min each. The dehydrated cells on the coverslip were coated with gold and observed under an SEM. For the analysis of the clearance activity, the *S. suis* SC197 suspension (1 × 10^6^ CFU/mL) was added to the well, incubate at 37 °C for 72 h, and washed three times with PBS. Subsequently, the protein was added to the wells at a final concentration of 200 μg/mL. The control group was treated with an equal amount of PBS as a negative control and incubated at 37 °C for 1 h. After the cells on the coverslip were washed with sterile PBS three times, they were fixed, dehydrated, sprayed with gold, and observed with an SEM.

### Evaluation of antibiofilm efficacy *in vivo*

A mouse biofilm model was constructed as described in previous studies, with minor modifications [48]. Specifically, the titanium plate was cut into small disks (diameter 6 mm × thickness 0.5 mm), disinfected, and placed on a 6-well plate. Two millilitres of the *S. suis* SC197 bacterial suspension (1 × 10^6^ CFU/mL) was added to each well and cultured at 37 °C for 72 h to promote biofilm formation. Titanium discs with biofilms were subsequently harvested to establish a mouse model of biofilm infection. Forty-eight female C57BL/6J mice were randomly divided into eight groups (n = 6): the control group (PBS treatment), Amp group (ampicillin treatment), Lys33795 group, Lys33795 + Amp group, GH25_muramidase group, GH25_muramidase+Amp group, CBD group, and CBD + Amp group. In accordance with standard surgical procedures, including anaesthesia, back hair removal, disinfection, and a sterile incision, a mouse biofilm-related infection model was established by subcutaneously implanting biofilm-coated titanium discs. As shown in Fig 7A, on postoperative Days 3 and 7, the treatment group received local injections of 100 μL of Lys33795 (2 mg/mL), GH25_muramidase (2 mg/mL), CBD (2 mg/mL), or the ampicillin suspension (10 mg/mL), while the control group received an equal amount of sterile PBS. On the 4th and 8th days after surgery, the combination therapy group received a local injection of 100 μL of the ampicillin suspension (10 mg/mL). All the mice were euthanized by carbon dioxide inhalation on the 10 th day. The obtained titanium sheets were subjected to SEM imaging. In addition, the titanium sheets were washed three times with sterile PBS and stained using the SYTO9/PI live/dead bacterial viability staining method. The biofilm was observed by acquiring images using a confocal CLSM (Zeiss Axio Observer Z1/7, Germany). In addition, the titanium sheets were washed 3 times with sterile PBS to remove planktonic bacteria. The titanium sheets were then shaken in sterile PBS until the biofilm was completely dissolved. The solution was then coated onto a solid plate for colony counting.

### Statistical analysis

All analyses were performed using GraphPad Prism 8.0 (Graph Pad Software, San Diego, CA). Data were analyzed using a student’s t test or one-way ANOVA and Tukey test, followed by least significant difference analysis. *P* < 0.05 was considered statistically significant. Data throughout the text and figures are presented as means ± standard deviations (SDs).

## Supporting information

S1(A) Fig SDS‒PAGE analysis of the purified of Lys33795, Lys8387 and Lys48548. (B) Spot test of the activity of Lys33795, Lys8387 and Lys48548 towards *S. suis* SC19 cultured on solid BHI agar.
(ZIP)

S2 Fig The stability of the antibacterial activity of Lys33795. The effects of temperature from 4 °C to 50 °C (A), pH from 2 to 10 (B), EDTA concentrations (C) and NaCl concentrations from 0 to 750 mM (D) on the activity of Lys33795 were detected. The maximum log_10_ CFU/mL reduction was regarded as the highest activity, and the ratio of the maximum reduction to the initial bacterial load was defined as 100% activity. The percentage of activity detected under each condition was compared with the highest activity to generate the values (% Rel. Activity) shown in this figure. The values are presented as the means ± SDs (*n* = 3). (E) Susceptibility of *S. suis* to Lys33795. Each strain was treated with 200 µg/mL Lys33795 or PBS for 1 h, the number of CFU in the sample was measured. The data are shown as the means ± SDs (n = 3).
(TIF)

S3 Fig Capsular images by HISS staining (capsule: blue purple, cells: dark blue). scale bars, 2 µm. (TIF)

S4 Fig Size-exclusion chromatography (GE Healthcare, Superdex 200 16/600 GL, buffer: 20 mM Tris-HCl, pH 7.5, 150 mM NaCl) of the GH25_muramidase (GH25_m) truncated fragment (A) and CBD truncated fragment (C). Both the GH25_muramidase and CBD truncated fragments were eluted at 93 mL (A and C). SDS‒PAGE analysis of the GH25_muramidase truncated fragment (B) and CBD truncated fragment (D).
(ZIP)

S5 Fig The structure prediction of the Lys33795 CBD dimer using AlphaFold3. The predicted ipTM and pTM values for this structure are 0.77 and 0.83, respectively.
(TIF)

S1 Table Lytic spectrum of Lys33795. (DOCX)

S2 Table The primers used in this study. (DOCX)
