# Supplementary material for: Dual activities of lysis and capsule degradation enable the bacteriophage protein Lys33795 to effectively disrupt Streptococcus suis biofilms
Source: PLoS Pathog. 2026 Oct 23;22(10):e1014741. doi: 10.1371/journal.ppat.1014741 (PMC13722301; doi:10.1371/journal.ppat.1014741)
Supplement: Supplementary file 1 [file ppat.1014741.s006.docx]

**Table S1. Lytic spectrum of Lys33795.**

| Strain number | Bacterial strain | Serotype | Spot test of Lys33795 (20 μg) | |
| --- | --- | --- | --- | --- |
|  |  |  | plaque | Halo |
| 1 | 052YH33 | 2 | + | + |
| 2 | CPD16-1 | 2 | - | - |
| 3 | CPD40 | 2 | + | + |
| 4 | CPD17 | 2 | + | + |
| 5 | CPD35 | 2 | + | + |
| 6 | SC19 | 2 | + | + |
| 7 | 1-302 | 2 | - | - |
| 8 | CPD41 | 2 | - | - |
| 9 | JZLQ022 | 2 | - | - |
| 10 | SC225 | 2 | + | + |
| 11 | P-1 | 2 | + | + |
| 12 | SC1483 | 3 | + | + |
| 13 | SC220 | 3 | - | - |
| 14 | SC295 | 3 | + | + |
| 15 | SC294 | 3 | + | + |
| 16 | SC140 | 3 | - | - |
| 17 | SC251 | 3 | - | - |
| 18 | SC1500 | 3 | + | + |
| 19 | SC197 | 3 | + | + |
| 20 | J2LQ001-7 | 7 | + | + |
| 21 | 242 | 7 | + | + |
| 22 | 2θ3 | 7 | + | + |
| 23 | SC15012 | 7 | + | + |
| 24 | SC493 | 9 | + | + |
| 25 | SC143 | 9 | + | + |
| 26 | SC457 | 9 | + | + |
| 27 | SC450-2 | 9 | + | + |
| 28 | SC74 | 9 | + | + |
| 29 | SC489 | 9 | + | + |
| 30 | SC498 | 9 | + | + |
| 31 | SC139 | 9 | + | + |
| 32 | SC442 | 9 | + | + |
| 33 | SC1502 | 9 | - | - |
| 34 | SC183 | 12 | + | + |
| 35 | SC198 | 12 | - | - |
| 36 | SC128 | 27 | + | + |

+ Lys33795 can form plaque or Halo; - Lys33795 can not form plaque or Halo.
