# Supplementary material for: Dual activities of lysis and capsule degradation enable the bacteriophage protein Lys33795 to effectively disrupt Streptococcus suis biofilms
Source: PLoS Pathog. 2026 Oct 23;22(10):e1014741. doi: 10.1371/journal.ppat.1014741 (PMC13722301; doi:10.1371/journal.ppat.1014741)
Supplement: Supplementary file 2 [file ppat.1014741.s007.docx]

**Table S2. The primers used in this study.**

| Primers | Sequence (from 5’ to 3’) |
| --- | --- |
| GH25_m-F | AGCAAATGGGTCGCGATGAAGAAAAACGATTATTTTATTG |
| GH25_m -R | TGGTGGTGGTGGTGCTTAGTCTAATAAAACAATGTTCTTG |
| CBD-F | AGCAAATGGGTCGCGGATGACGCAACAACTACTACACAAT |
| CBD-R | TGGTGGTGGTGGTGCTCAAATTTTCTTCAATTGCATACCT |
| GH25_m-E1-F | AGCAAATGGGTCGCGATGAAGAAAAACGATTATTTTATTG |
| GH25_m-E1-R | GCTCACGGGGGTTCTAGAACCCCCGTCTAATAAAACAATGT |
| GH25_m-E2-F | TTAGACGGGGGTTCTAGAACCCCCGTGAGCAAG |
| GH25_m-E2-R | TGGTGGTGGTGGTGCCTACTTGTACAGCTCGTCCATG |
| CBD-E1-F | AGCAAATGGGTCGCGGATGACGCAACAACTACTACACAAT |
| CBD-E1-R | GCTCACGGGGGTTCTAGAACCCCCAATTTTCTTCAATTGCA |
| CBD-E2-F | AAAATTGGGGGTTCTAGAACCCCCGTGAGCAAG |
| CBD-E2-R | TGGTGGTGGTGGTGCCTACTTGTACAGCTCGTCCATG |
| D9-F | AACGATTATTTTATTGCTGTATCGGCTTATCAA |
| D9-R | TTGCTAATAAAATAACGACATAGCCGAATAGTT |
| D92-F | CGCTATCTGGTCTGTGCCTACGAGGACAGCGCA |
| D92-R | GCGATAGACCAGACACGGATGCTCCTGTCGCGT |
| E94-F | CTGGTCTGTGACTACGCGGACAGCGCAAGTAGC |
| E94-R | GACCAGACACTGATGCGCCTGTCGCGTTCATCG |
| Y125-F | TACGAGCCTATCTACGCCAGCTATAAGCCGTTT |
| Y125-R | ATGCTCGGATAGATGCGGTCGATATTCGGCAAA |
| S184-F | TGGTGGCAATTCACCGCAACCGCTTTACAAGGC |
| S184-R | ACCACCGTTAAGTGGCGTTGGCGAAATGTTCCG |
| G189-F | TCAACCGCTTTACAAGCCGGACTAGACAAGAAC |
| G189-R | AGTTGGCGAAATGTTCGGCCTGATCTGTTCTTA |
| T220-F | CTACCCATGGAATTCGCATTTCAAATCTCTGGC |
| T220-R | GATGGGTACCTTAAGCGTAAAGTTTAGAGACCG |
| Primers | Sequence (from 5’ to 3’) |
| G242-F | GCCAATACGGCACGTGCTATTTATCGAGGGTTG A |
| G242-R | CGGTTATGCCGTGCACGATAAATAGCTCCCAAC |
| I243-F | AATACGGCACGTGGTGCTTATCGAGGGTTGACT |
| I243-R | TTATGCCGTGCACCACGAATAGCTCCCAACTGA |
| Y244-F | ACGGCACGTGGTATTGCTCGAGGGTTGACTCAT |
| Y244-R | TGCCGTGCACCATAACGAGCTCCCAACTGAGTA |
| R245-F | GCACGTGGTATTTATGCAGGGTTGACTCATATT |
| R245-R | CGTGCACCATAAATACGTCCCAACTGAGTATAA |
| D251-F | GGGTTGACTCATATTGCTGAATGGAATGTTATT |
| D251-R | CCCAACTGAGTATAACGACTTACCTTACAATAA |
| G265-F | TATAAAGACTCAACTGCTCGAGACATGCCACAC |
| G265-R | ATATTTCTGAGTTGACGAGCTCTGTACGGTGTG |
| W273-F | ATGCCACACTATAAGGCGGACGCTAAGGCGCCT |
| W273-R | TACGGTGTGATATTCCGCCTGCGATTCCGCGGA |
| P278-F | TGGGACGCTAAGGCGGCTTGGTATACTCGTGCT |
| P278-R | ACCCTGCGATTCCGCCGAACCATATGAGCACGA |
| W279-F | GACGCTAAGGCGCCTGCGTATACTCGTGCTATT |
| W279-R | CTGCGATTCCGCGGACGCATATGAGCACGATAA |
| R282-F | GCGCCTTGGTATACTGCTGCTATTGTAGGTATG |
| R282-R | CGCGGAACCATATGACGACGATAACATCCATAC |
| Lys3M-F | CAATCAAACCAAAATTTACCAGGACTACCCCTTGAATTCACATTTCAAATC |
| Lys3M-R | GATTTGAAATGTGAATTCAAGGGGTAGTCCTGGTAAATTTTGGTTTGATTG |
|  |  |
|  |  |
|  |  |
|  |  |
|  |  |
|  |  |
|  |  |
|  |  |
|  |  |
|  |  |
|  |  |
|  |  |
|  |  |
|  |  |
|  |  |
|  |  |
|  |  |
|  |  |
|  |  |
|  |  |
|  |  |
|  |  |
|  |  |
|  |  |
|  |  |
|  |  |
